# A Systematic Review of Cyber Threat Intelligence: The Effectiveness of Technologies, Strategies, and Collaborations in Combating Modern Threats

**DOI:** 10.3390/s25144272

**Published:** 2025-07-09

**Authors:** Pedro Santos, Rafael Abreu, Manuel J. C. S. Reis, Carlos Serôdio, Frederico Branco

**Affiliations:** 1 Department of Engineering, School of Sciences and Technology, University of Trás-os-Montes e Alto Douro, 5000-801 Vila Real, Portugal; rubenpedro32@gmail.com (P.S.); rabreu@utad.pt (R.A.); cserodio@utad.pt (C.S.); 2 Engineering Department, Institute of Electronics and Informatics Engineering of Aveiro (IEETA), University of Trás-os-Montes e Alto Douro, 5000-801 Vila Real, Portugal; mcabral@utad.pt; 3 Algoritmi Center, University of Minho, 4710-057 Braga, Portugal; 4 INESC TEC-Institute for Systems and Computer Engineering, Technology and Science, Rua Dr. Roberto Frias, 4200-465 Porto, Portugal

**Keywords:** cybersecurity, cyber threat intelligence, frameworks, AI, machine learning, platforms, advanced persistent threats

## Abstract

Cyber threat intelligence (CTI) has become critical in enhancing cybersecurity measures across various sectors. This systematic review aims to synthesize the current literature on the effectiveness of CTI strategies in mitigating cyber attacks, identify the most effective tools and methodologies for threat detection and prevention, and highlight the limitations of current approaches. An extensive search of academic databases was conducted following the PRISMA guidelines, including 43 relevant studies. This number reflects a rigorous selection process based on defined inclusion, exclusion, and quality criteria and is consistent with the scope of similar systematic reviews in the field of cyber threat intelligence. This review concludes that while CTI significantly improves the ability to predict and prevent cyber threats, challenges such as data standardization, privacy concerns, and trust between organizations persist. It also underscores the necessity of continuously improving CTI practices by leveraging the integration of advanced technologies and creating enhanced collaboration frameworks. These advancements are essential for developing a robust and adaptive cybersecurity posture capable of responding to an evolving threat landscape, ultimately contributing to a more secure digital environment for all sectors. Overall, the review provides practical reflections on the current state of CTI and suggests future research directions to strengthen and improve CTI’s effectiveness.

## 1. Introduction

Cyber threat intelligence (CTI) has become a cornerstone of modern cybersecurity, allowing organizations to anticipate threats and act proactively. With the increasing complexity of digital systems and the constant evolution of attack techniques, traditional reactive approaches have become insufficient to protect critical assets. In this context, CTI stands out as an essential strategy that combines the collection, analysis, and sharing of threat information, enabling organizations to respond quickly and accurately to emerging risks.

A key component of CTI is the use of machine learning algorithms, which have revolutionized threat detection by automating the analysis of large volumes of data. Algorithms such as Support Vector Machines (SVMs) and convolutional neural networks (CNNs) can identify patterns and anomalies indicative of suspicious activity with significantly higher accuracy than traditional signature-based methods [1]. Moreover, the adaptability of machine learning models makes them particularly effective at detecting advanced threats, such as Advanced Persistent Threats (APTs), which often employ sophisticated techniques to evade conventional detection.

Secure information sharing between organizations is a fundamental aspect of CTI’s effectiveness. Blockchain technology has been offered as a possible solution to this dilemma due to its decentralized and tamper-resistant nature. Immutable records provide trust to organizations working outside their jurisdictions, particularly in cases where coordinated responses are essential to mitigating global cyber threats [2].

An additional determining factor of CTI is Open-Source Intelligence (OSINT), which delivers real-time data gathered from publicly available data on platforms such as social media, forums, or websites. When properly integrated, OSINT enhances overall threat detection capabilities, which supports early detection of Indicators of Compromise (IoCs). However, there are still challenges related to the quality and relevance of collected data, which must be addressed to ensure accurate threat analysis [3].

Table 1 below illustrates the main applicable technologies used in CTI as well as their applications and possible challenges.

The effectiveness of CTI also depends on overcoming persistent challenges, such as the dynamic nature of cyber threats, which requires continuous adaptation of detection strategies, and the high volume of data, which complicates the distinction between real risks and false positives. Optimization techniques, such as metaheuristic algorithms, have been investigated to enhance the selection of relevant features in threat detection, thereby reducing false positives and improving the efficiency of security systems.

The topics discussed, from advances in machine learning and the use of blockchain for intelligence sharing to integrating OSINT and applying optimization techniques, highlight the multifaceted nature of cyber threat management. Several previous reviews discussed in this paper have already examined the domain of cyber threat intelligence (CTI), focusing on specific aspects such as artificial intelligence-based detection techniques, information sharing platforms, and the use of Open-Source Intelligence (OSINT) from sources like the dark web to anticipate threats. For instance, Ref. [2] conducted a thorough review of blockchain and artificial intelligence (AI) technologies and demonstrated how blockchain helps to enhance AI with data integrity and decentralization, covering blockchain architectures and consensus mechanisms. Although they provided a detailed overview of blockchain for AI applications, they primarily focused their review on definitions of technical infrastructure and applications, with limited information about how these initiatives could be used in operational CTI ecosystems. Ref. [4] outlined the functions of CTI to enable organizational cybersecurity resilience, proposing a multi-layered CTI framework consisting of CTI knowledge bases, detection models, and visualization dashboards. However, they only provided methods of IT implementation at enterprise levels of practice, without an ability to compare the depth of strategy effectiveness, collaborative models, and technology improvements during implementation across the various sectors, leaving OSINT and optimization techniques not covered. The authors from [5] reviewed CTI and blockchain technologies. They analyzed 47 studies over the last five years, categorizing studies by CTI lifecycle phases, levels of CTI, types of blockchain, and supporting technologies. While their work offered a vast number of blockchain-supported mechanisms, it had major limitations on the discussion surrounding the technical integration of blockchain to CTI data sharing, failing to explore machine learning advancements or policy-driven CTI models. Lastly, Ref. [1] provided a broader review of machine learning and data mining techniques applied within intrusion detection, but due to its publication date, it omitted many recent and novel advances on more recent AI, threat prioritization, and blockchain for secure information sharing.

While these reviews offer valuable insights, they present significant limitations, often addressing the topic in a fragmented way, concentrating on narrow contexts or particular technological approaches without providing an integrated and comprehensive view of the subject. Furthermore, due to the rapid evolution within the cybersecurity field, many existing reviews have become outdated, lacking recent advancements such as the use of blockchain for secure information sharing or the application of optimization techniques to reduce false positives in detection systems, which remain very important. The current systematic review seeks to address these gaps by providing an up-to-date perspective by integrating diverse and emerging technologies, tools, and methodologies, demonstrating a significant evolution in this field. It examines the intersection of machine learning, blockchain-based intelligence sharing, OSINT integration, and the role of optimization techniques in CTI. Therefore, it contributes to a deeper understanding of contemporary challenges in CTI and offers clear directions for future research. This systematic review examines these aspects in detail, providing in-depth knowledge of the field’s current challenges and emerging solutions. Addressing these complex issues reinforces the need for continuous innovation in CTI practices, which are becoming increasingly critical for ensuring a robust and resilient security posture.

### Contributions

**Figure 1:**
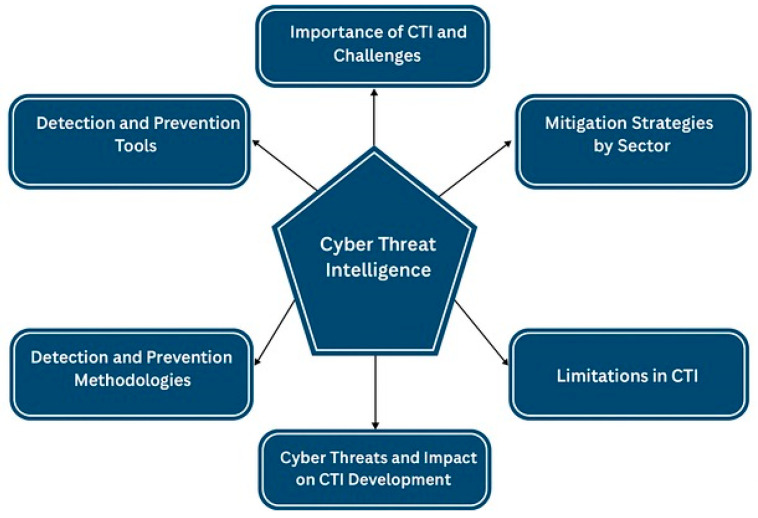
Structure of the study and main topics addressed.

This systematic review was conducted to compile and investigate mitigation strategies applied in various sectors. In addition, the review also examines detection and prevention tools, methodologies, and the limitations and challenges involved in developing cyber threat intelligence (CTI), providing a solid foundation for organizations aiming to adopt best practices in cybersecurity. These insights guide the present study by identifying key areas that require further research or updated strategies, which are discussed in detail in the results section. The structure of this work is outlined in Figure 1.

In the results section, several essential topics are addressed, as outlined in Figure 1. Each component is fundamental to a comprehensive understanding of cyber threat intelligence (CTI).

The section begins by discussing the importance of CTI and the intrinsic challenges it faces in this domain. One of the main obstacles is the constant evolution of cyber threats, which requires cyber intelligence to adapt swiftly to protect organizations effectively. We explore the indispensable role of CTI in enhancing organizational security and discuss the difficulties and challenges associated with implementing these strategies. Additionally, we examine how different sectors adjust their security practices to meet specific operational needs and contexts.

Furthermore, the detection tools used to identify and neutralize threats before they cause significant damage are considered. We investigate the most used tools, their functionalities, and how they have evolved to keep pace with the dynamic threat landscape.

Building upon this, the review explores detection and prevention methodologies, emphasizing how these methodologies contribute to attack mitigation and identify existing limitations. Understanding these gaps and challenges is crucial for guiding future research and promoting continuous advancements in this field.

This systematic review aims to bridge the knowledge gaps by addressing the following research questions:

- RQ1: How effective are cyber threat intelligence (CTI) strategies in mitigating cyberattacks in different sectors?
- RQ2: Which tools should be used to combat these attacks efficiently?
- RQ3: Which analysis methodologies are most effective in detecting and preventing cyber threats through CTI?
- RQ4: What are the limitations of current CTI approaches, and what strategies can be implemented to overcome them?
- RQ5: What are the emerging cyber threats that may impact the future development of CTI?

By answering the first research question, we aim to find out why it is important to evaluate CTI strategies across the referred sectors. By answering RQ2, we explain why the tools matter and what the challenge is in selecting the appropriate tool. The third research question addresses the importance of analysis methodologies and what distinguishes one from another in terms of their effectiveness. RQ4 tries to bridge the gap about the known limitations in current CTI approaches and why mitigating them is crucial. Lastly, RQ5 aims to identify emerging threats that help shape the future of CTI evolution.

## 2. Materials and Methods

The methodology of this systematic review was carefully structured to ensure comprehensive coverage of cyber threat intelligence (CTI). Following the PRISMA 2020 guidelines, the process involves several steps to identify, select, and analyze the most relevant studies. Table 2 outlines the overall methodological process described in this section to facilitate understanding of the phases.

This section presents the methodology employed in conducting the analysis, including research questions, search strategy, selection criteria, keywords, databases, and data extraction procedures. The research protocol is presented in Table 3 below:

### 2.1. Identification

In this initial phase, a systematic search was conducted in the selected academic databases to identify a comprehensive list of potentially relevant cyber threat intelligence (CTI) studies. The databases chosen for this review were IEEE Xplore, Science Direct, Scopus, and Web of Science. These databases were selected as they provide large coverage of peer-reviewed literature that is specifically relevant to the technological and scientific fields related to cyber-threat intelligence and cybersecurity. They index an extensive range of high-impact journal articles and conference proceedings related to this research area; therefore, a more comprehensive data collection can be ensured from these databases. Unlike larger literature searching platforms such as Google Scholar, these databases provide more focused and curated content, which enhances the quality and relevance of the studies included in this review. The search was performed on 1 January 2025 using a standardized search string to ensure consistency across all databases.

#### Search String

The exact search string was used to guarantee the consistency of the results obtained in the selected databases for this systematic review. The search string used was:

(“Cyber Threat Intelligence” OR “CTI”) AND (“cybersecurity” OR “information security” OR “cyber attacks” OR “threat mitigation” OR “cti tools”).

### 2.2. Screening

In this phase, the titles and abstracts of the identified studies were screened to ensure that only publications meeting the predefined inclusion criteria were considered for the next stage. Two independent reviewers screened the studies for inclusion. Any disagreements found were resolved through discussion, and a third reviewer was consulted if a disagreement was still not resolved between the two reviewers.

The articles were then evaluated according to the following inclusion and exclusion criteria.

#### 2.2.1. Inclusion Criteria

Specific inclusion criteria were established to ensure that the review includes only studies relevant to the topic under investigation. These aim to select publications contributing to understanding the subject under analysis, ensuring that the final selection consists of high-quality, pertinent studies.

The inclusion criteria defined for this systematic review are as follows:

- Studies that approach concepts related to CTI;
- Publications that meet the key terms and search criteria previously defined.

#### 2.2.2. Exclusion Criteria

The initial search retrieved a wide range of scientific publications, including some irrelevant to the study conducted in this systematic review. Therefore, an exclusion process was applied to remove publications that did not align with the research objectives.

The exclusion criteria aim to eliminate publications that do not fit this review’s scope. Duplicate, outdated, and/or incomplete studies were also excluded. The following exclusion criteria are defined and applied in the following order:

- The publication is a duplicate.
- The article is not written in English.
- The study was published before 2018.
- The publication record does not correspond to a complete paper.
- The full version of the article is unavailable.
- The study does not align with the scope of the RQs.

As a result of this screening process, 1582 articles were initially identified, of which 684 were excluded for being duplicates. This left 898 articles for the eligibility analysis.

### 2.3. Eligibility

The 898 non-duplicate articles were evaluated during the initial phase based on their titles and abstracts. This process excluded 745 articles that did not meet the predefined inclusion criteria. Consequently, 153 articles were selected for a full-text analysis.

After applying the inclusion and exclusion criteria to the full texts, 70 articles were excluded because they did not meet the requirements, and 40 were removed due to unavailability of full-text access. At the end of this phase, 43 articles met all the established criteria and were included in the systematic review, forming the foundation for the final analysis. Although this number may seem moderate, it is well aligned with similar systematic reviews in the field of cyber threat intelligence (CTI), where final selections typically range between 30 and 50 studies. Consequently, the 43 selected studies reflect a carefully compiled and high-quality dataset resulting from a structured search strategy across four databases (IEEE Xplore, ScienceDirect, Scopus, and Web of Science) and the rigorous application of clearly defined inclusion, exclusion, and quality assessment criteria, ensuring the necessary relevance for meaningful comprehension of the current CTI landscape.

### 2.4. Synthesis

The 43 selected studies were examined and integrated in the synthesis phase to identify patterns, gaps, and trends in cyber threat intelligence. The articles were reviewed using a quality checklist to ensure that publications with relevant and high-quality information were included in the conclusions.

#### Quality Checklist

A quality checklist was defined to assess the quality of the articles included in the sample (see Table 4). This checklist ensured that only studies containing the necessary metadata and addressing the RQs presented were retained for final analysis. The clarity with which an article presents its contents and the justification provided for each step taken are essential factors in determining its quality. Therefore, articles that support all their viewpoints with solid examples or well-founded arguments are valued. The quality checklist is presented in Table 4 below.

The following table summarizes the assessment of the articles selected for this systematic review using key questions to analyze critical aspects of cyber threat intelligence (CTI) approaches. Each article was evaluated based on its coverage of essential topics, including the effectiveness of threat mitigation strategies, recommended tools for mitigating attacks, analysis methodologies, limitations of current approaches, and identification of emerging threats. This assessment provides a comprehensive perspective on each study’s contribution to advancing CTI practices. The detailed assessment results are presented in Table 5 below, which summarizes how each study met the quality criteria.

**Figure 2:**
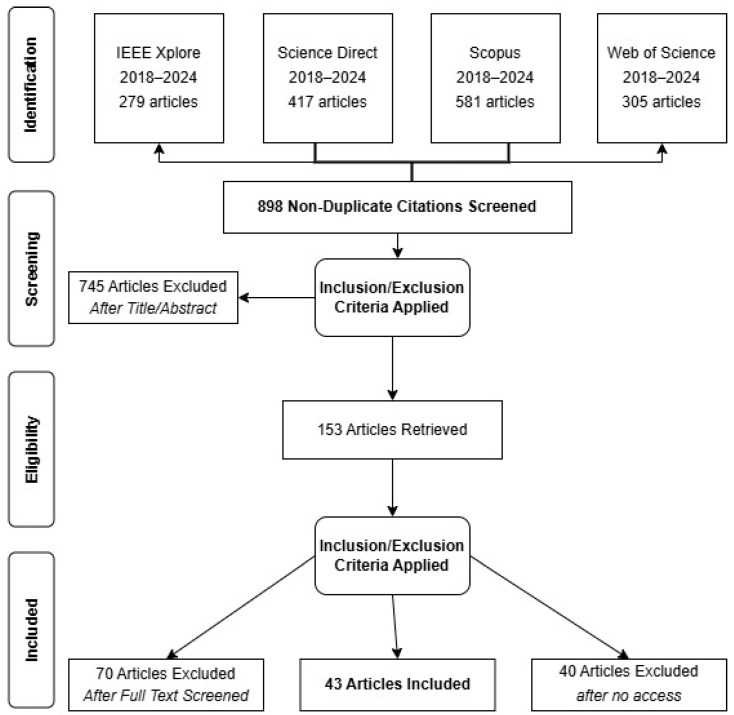
PRISMA review process flow.

The following image presents the PRISMA flow diagram, which clearly illustrates the article selection process for this systematic review. This diagram is essential for highlighting the steps followed, from the initial identification of studies to the final inclusion of articles, while also highlighting the criteria applied at each stage and demonstrating the transparency of the review process. This process is illustrated in Figure 2, following the PRISMA 2020 flow diagram, detailing the stages of identification, screening, eligibility, and final inclusion.

## 3. Results

### 3.1. CTI Integration Frameworks and Platforms

Integrating cyber threat intelligence (CTI) into organizational security infrastructure has become essential as cyber threats evolve and increase in complexity. Various innovative frameworks have emerged in response to these challenges, each offering distinct approaches to proactively process and utilize CTI data. This section examines multiple solutions, detailing their methodologies, advantages, and limitations, while highlighting recent advancements in the field and suggesting improvements to address current challenges.

Guo et al. [6] proposed a framework that leverages BERT and deep learning to unify structured and unstructured data from diverse CTI sources. The framework significantly improved the precision in entity identification by employing an attention mechanism, while a modified Levenshtein algorithm facilitated efficient data fusion. The resulting cybersecurity knowledge graph (CKG) supports the detection of Advanced Persistent Threats (APTs) and zero-day attacks by providing a comprehensive view of the threat landscape. Nonetheless, the system’s reliance on a constant influx of data poses challenges. As the volume of heterogeneous information grows, more adjustments and larger resource allocations become necessary, which may impact the system’s overall performance.

Complementing this line of work, Gao et al. [7] introduced THREATRAPTOR, a platform that integrates natural language processing (NLP) with a custom query language (TBQL) to process extensive amounts of Open-Source Cyber Threat Intelligence (OSCTI). Its strength lies in the capacity to detect subtle behavioral patterns, often unnoticed by conventional audits. By employing TBQL, organizations can flexibly adapt the system to varied contexts, illustrating the potential of customizable CTI tools. While Guo et al. [6] emphasize structured data through knowledge graphs, THREATRAPTOR identifies patterns from unstructured data. A hybrid approach combining both could further strengthen threat detection in domains such as finance, where structured data (e.g., transactions) and unstructured data (e.g., suspicious emails) are crucial.

In the context of privacy and decentralization in IoT environments, El Jaouhari and Etiabi [8] addressed these challenges by proposing FedCTI, a framework that decentralizes the training of machine learning models. Rather than aggregating all data in a centralized location, FedCTI processes information directly on individual devices, thus alleviating privacy risks. The framework demonstrates how distributed intelligence can offer a viable alternative for IoT ecosystems by questioning the necessity of centralized data for practical threat analysis. However, ensuring model consistency across multiple devices remains a significant challenge, potentially leading to disparities in detection accuracy. Achieving a balance between data protection and system uniformity is critical for sensitive environments like hospitals, where connected medical devices are widely used. Periodic model updates and the adoption of standardized communication protocols can help address these limitations.

Another relevant contribution comes from Shin et al. [9], who employed GloVe embeddings to map tactics, techniques, and procedures (TTPs) within the MITRE ATT&CK framework. They proposed the Tactic Match Rate (TMR) metric, which achieved up to 96% accuracy when correlating concurrent tactics across sophisticated attacks. Nevertheless, the framework struggled with TTPs that could belong to multiple tactics, thereby reducing its effectiveness against more complex threats. This limitation could be addressed by integrating NLP-based approaches, such as those used in THREATRAPTOR, to better manage dynamic patterns and ambiguities in TTP classification.

Additionally, Aldhaheri et al. [10] explored deep learning techniques for intrusion detection in IoT environments, employing convolutional neural networks (CNNs), Recurrent Neural Networks (RNNs), and Long Short-Term Memory (LSTM) models. While this framework addresses the challenge of processing large and diverse data streams, its reliance on large labeled datasets presents a significant challenge. Securing such datasets is costly and time-consuming, reflecting a broader challenge in CTI: scaling data labeling and annotation to keep pace with rapidly evolving threats. Potential solutions include using self-learning algorithms or adopting federated data models, such as those proposed by FedCTI [8].

Lastly, Alam et al. [11] introduced the LADDER framework, which extracts attack patterns from CTI reports and maps them into the MITRE ATT&CK framework. By employing BERT for malware behavior prediction and constructing knowledge graphs to support threat analysis, LADDER achieved high accuracy in forecasting attack patterns across multiple platforms, including Android. However, the framework’s reliance on deep learning underscores the need for adaptability in dynamic environments, where training data can quickly become obsolete. Continuous model updates and real-time behavioral analysis can help to mitigate this limitation. Table 6 provides a summary of these frameworks, outlining their key methodologies, advantages, and limitations.

### 3.2. Behavioral Analysis Techniques in Threat Detection

As cyber threats become more sophisticated, machine learning (ML) and behavioral analysis have become essential tools for identifying and mitigating malicious activities. Unlike traditional approaches, which often fail to recognize subtle, evolving threats, ML-based systems and behavioral analysis can effectively identify patterns and behaviors by learning from large volumes of historical data. This enables the automatic detection of complex attacks, including zero-day exploits; unknown malware; and persistent, stealthy attacks.

Noor et al. [12] proposed an automated framework that leverages ML for threat detection in the financial sector, focusing on high-level Indicators of Compromise (IOCs) such as tactics, techniques, and procedures (TTPs); malware; and attack tools. Utilizing natural language processing (NLP), the study extracted attack patterns from unstructured cyber threat intelligence (CTI) reports, organizing them into different threat group profiles. Attribution was performed using machine learning models, such as Deep Learning Neural Networks (DLNNs), which excel in identifying complex events. However, the framework’s reliance on textual data was limited, particularly in scenarios involving overlapping data, such as attacks attributed to the Copykittens group, known for its sophisticated phishing techniques. While this model enhances automated threat attribution and offers proactive defense, challenges remain regarding data quality and integrating heterogeneous CTI sources.

Complementing Noor et al.’s approach, Husák et al. [13] employed a hybrid methodology that combines data mining, entity reputation analysis, and time series analysis to predict malicious activities. This integration improved prediction accuracy by identifying recurring attack patterns and forecasting spikes in security incidents. The study also emphasized the importance of predictive methods such as attack projection, reputation-based forecasting, and time series analysis. However, the requirement for large volumes of data for training and the dependence on data quality still represent considerable challenges. Together, the approaches [12,13] suggest that integrating textual data with additional data sources can improve the precision and coverage of threat detection models.

Tang et al. [14] employed an innovative strategy to detect Advanced Persistent Threats (APTs) by combining knowledge graphs with deep learning techniques, including Graph Neural Networks (GNNs) and convolutional neural networks (CNNs). These techniques enable the mapping of suspicious behaviors within large volumes of unstructured data, leveraging the integration of graphs and deep learning to model and respond to APT threats. Ontology modeling and graph-based reasoning, such as Graph Neural Networks (GNN) and Graph Convolutional Networks (GCN), further enhance the correlation of complex behaviors.

To protect industrial control systems (ICSs), which are frequent targets of persistent attacks due to their critical nature, Imran et al. [15] applied Random Forest algorithms and data balancing strategies. Their study used the Synthetic Minority Over-sampling Technique (SMOTE) to address class imbalance, improving detection accuracy up to 99%. Deep learning models, such as RNN and LSTM, were also evaluated, achieving an accuracy of 97%. The MITRE ATT&CK framework supported threat modeling, highlighting the benefit of combining various machine learning approaches and security frameworks to enhance protection in critical industrial environments. The use of SMOTE was crucial to improving model performance with imbalanced datasets.

Using the NIST framework, which categorizes cybersecurity practices into five core functions (Identify, Protect, Detect, Respond, and Recover), Kaur et al. [16] analyzed AI applications in cybersecurity, finding that 60% of 236 articles focused on the “Detect” function, primarily leveraging ML methods for intrusion detection and near real-time security monitoring. Despite this emphasis, the study also identified several limitations: outdated or limited datasets, a lack of real-time alert automation, and a lack of transparency in AI models. Emerging directions, such as predictive intelligence and automated honeypots, offer promising potential, although integrating heterogeneous data sources remains a challenge.

Kante et al. [17] investigated mitigating ransomware attacks by combining cyber threat intelligence (CTI) with ML algorithms. Their study highlighted that these combined approaches significantly enhance ransomware detection and mitigation capabilities, although the rapid evolution of these threats requires continuous updates to both models and training data. The authors emphasized the importance of adaptive models and collaborative intelligence networks for proactive responses to complex attacks. When combined with honeypots, methods such as Decision Trees and Random Forests showed a reduced false positive rate in ransomware detection. Still, these methods rely heavily on the availability of large volumes of labeled data. This point reinforces the findings of Kaur et al. [16], highlighting the need for more explainable AI models.

Focusing on ransomware detection and classification in fog computing environments, Homayoun et al. [18] introduced DRTHIS, a hybrid solution that combines LSTM (Long Short-Term Memory) and CNN (convolutional neural networks) to distinguish ransomware from legitimate software on IoT devices. Fog computing, characterized by a decentralized architecture that processes data closer to the source, enables faster threat response. DRTHIS achieved 99.6% accuracy with a 0% false positive rate. The integration of LSTM, which captures temporal patterns, and CNN, which detects spatial patterns, proved essential for identifying new ransomware attacks, such as CryptoWall and Sage. This low false positive rate positions DRTHIS as a highly effective solution for resource-constrained environments.

In response to the growing complexity of cyber threats and their dispersion across open sources such as the dark web, Cherqi et al. [19] proposed ConGAN-BERT, a semi-supervised threat detection model that combines generative adversarial networks (GANs), BERT, and contrastive learning. This integration reduces the reliance on large volumes of labeled data, a notable advantage in environments with sparse or noisy data. This semi-supervised approach was advantageous when labeled datasets were limited. This aligns with prior studies emphasizing data scarcity in detection models, such as those by Kante et al. [17].

Focusing on Intrusion Detection Systems (IDSs) within darknet environments, Pour and Bou-Harb [20] evaluated the performance of two IDS tools, Bro and Snort, against threats such as botnets and worms. The analysis considered the probing rate, detection time, and the number of IP addresses required for adequate identification. Bro demonstrated superior performance compared to Snort in handling IPv6 traffic and stealthy attacks, whereas Snort was more effective in IPv4 scenarios but required a larger number of IP addresses for detection. Both systems exhibited limitations when detecting large-scale attacks involving numerous distributed devices. These findings suggest the importance of tailoring IDS configurations to specific network environments and traffic patterns to enhance their detection capabilities.

Xiao [21] developed the IoT-CTIS system, which uses machine learning to detect malware in IoT devices through a three-layer architecture. The IoT layer collects device data, the Edge layer performs initial processing, and the Cloud layer conducts final processing and predictive analysis. The model uses both supervised and unsupervised learning, integrating natural language processing (NLP) for IOC extraction and autoencoders to reduce data complexity. Threats, like DDoS botnets, were detected with high precision and recall, outperforming models like SVM and CNN. The study highlighted how this combination of techniques enhances IoT network security by reducing false positives and negatives.

In the Windows ecosystem, which is frequently targeted by diverse malware threats, Huang et al. [22] developed MAMBA, which leverages the MITRE ATT&CK framework alongside deep neural networks and attention mechanisms to map TTPs to specific API calls. Organized into three phases (data collection, mapping between resources and malware execution, and predictive analysis), MAMBA distinguishes itself through high accuracy in TTP identification and providing detailed explanations of malicious behaviors. However, continuous updates to the MITRE framework must remain current, underscoring the rapid evolution of attacker techniques. The study demonstrated the value of integrating established threat intelligence frameworks with advanced deep learning to support detailed forensic analysis.

In the context of entity extraction for CTI, Chang et al. [23] proposed a unified model that combines CySecBERT—an adaptation of BERT for cybersecurity—with convolutional neural networks (CNNs) to address challenges such as discontinuous entity extraction and data imbalance. This approach significantly improved precision and recall, facilitating the development of more Comprehensive Knowledge Graphs (CKGs), vital for systematically correlating threat information. By building more accurate CKGs, analysts can better link disparate threat indicators and strengthen overall threat detection.

K. Zhang et al. [24] reviewed Named Entity Recognition (NER) techniques for recognizing entities in unstructured texts. Deep learning-based models such as BiLSTM-CRF and BERT were particularly effective in capturing context and resolving ambiguity. The review also identified transfer learning as a solution to improve model performance with limited data. Additionally, integrating GANs to generate labeled data was highlighted to address data scarcity. Zhang et al. emphasized that although models like BERT and RoBERTa present advancements, there are still challenges, such as creating high-quality labeled datasets and semantic ambiguity.

To address the specialized requirements of cybersecurity, Park and You [25] developed CTI-BERT, a language model trained from scratch using cybersecurity-focused vocabulary. It outperforms general-purpose models, which often struggle with technical terminology related to threats. CTI-BERT was trained on sources such as MITRE ATT&CK, CVE, and CAPEC, thereby improving performance in entity recognition and TTP classification tasks. The model demonstrated effectiveness in enhancing cybersecurity operations and threat detection.

Lastly, in a complementary effort to add structure to the identification and classification of cyberattacks, Trifonov et al. [26] integrated the Cyber Kill Chain model with an established threat taxonomy, offering greater clarity in mapping malicious events. While this framework provides a more systematic approach for examining multiple phases of an attack, the rapid evolution of illicit techniques, such as Crimeware-as-a-Service (CaaS), remains a critical challenge. The study concluded that maintaining up-to-date taxonomies and AI models is essential for keeping pace with advancements in the cyberthreat arena, where malicious actors’ creativity often outstrips existing defenses. Table 7 summarizes the results and advantages of each study, offering a comparative view of each contribution across the literature.

### 3.3. CTI Sharing and Collection Platforms for CTI

Platforms for collecting and sharing cyber threat intelligence (CTI) play a crucial role in defending against cyberattacks. These platforms enhance organizational responsiveness and support threat identification by enabling real-time exchange of threat information. They contribute to attack prevention by fostering collaboration among companies and governmental entities. This section provides an overview of key CTI platforms, highlighting their methodologies, strengths, and limitations and comparing the different approaches adopted across the reviewed studies.

Focusing on open-source threat intelligence (OSCTI) automation, Gao et al. [27] introduced SecurityKG, an automated platform that leverages ML and NLP to construct a cybersecurity knowledge graph. This approach allows for the visualization and analysis of adversarial tactics, thereby improving threat detection and incident response. However, the study notes that real-world deployment may encounter obstacles to integrating existing systems.

Daou et al. [29] and Van Kranenburg et al. [33] proposed complementary CTI approaches tailored for small and medium-sized enterprises (SMEs), each with distinct features [29]. They developed an OSINT-based CTI platform hosted on AWS using MongoDB for malware analysis and Power BI for visualization. This solution is cost-effective and well-suited for SMEs with limited resources, although it may lack analytical depth compared to more advanced solutions. In contrast, Ref. [33] introduced a peer-to-peer network for sharing risk information, using graph analysis to contextualize and prioritize threats collaboratively. However, this model requires continuous monitoring of data security and privacy. A hybrid solution combining both approaches could meet the needs of these companies.

To address trust in CTI sharing, Wagner et al. [31] evaluated 30 CTI-sharing platforms and found that only four supported manual and automatic trust mechanisms, while most relied on non-transparent processes. Initial testing revealed that new participants struggled to gain trust, but their reputation improved through consistently sharing high-quality CTI. On the other hand, contributors exhibiting negligent or malicious behavior lost credibility, resulting in restricted access to critical information. The system prioritized helpful contributions to maintain the reliability of the information. The study also highlighted that account hijacking significantly reduces trust, necessitating the implementation of recovery measures. Additionally, sharing information beyond the trust circle triggered automatic alerts and sanctions. These findings underscore the need for transparent processes and robust reputation mechanisms to ensure secure and collaborative CTI sharing.

In another study, Bou-Harb et al. [32] developed preprocessing models to sanitize vast datasets collected from millions of IP addresses within darknet networks. Using a probabilistic approach, the system filtered out invalid traffic with zero false negatives. This enabled the detection of high-intensity attacks (above 200,000 Mbps), such as DDoS, ensuring higher data quality for analysis. Additionally, time-series techniques were employed to identify coordinated probing campaigns, achieving 98% accuracy and outperforming the previous processing time of the methods. While it demonstrated high effectiveness, its success depends on the quality of incoming data and its adaptability to evolving attack vectors. This method enhances cyber situational awareness and facilitates the identification and mitigation of large-scale threats.

Nguyen et al. [28] proposed a blockchain-based platform that leverages smart contracts to promote decentralized threat information sharing. This approach eliminates single points of failure and preserves data privacy, an essential feature for critical infrastructure environments, such as power plants. However, the complexity and implementation costs present barriers to its widespread adoption. On the other hand, X. Zhang et al. [30] focused on the quality of shared information by incorporating a Proof of Reputation (PoR) algorithm to verify data reliability, preventing the spread of false information. This mechanism encourages safer collaboration in sectors with stringent security requirements. However, the effectiveness of the PoR approach relies heavily on consistent participation and compliance by contributing organizations, which may limit its appeal or scalability.

Breaking new ground in cybersecurity for the energy sector, Pahlevan et al. [35] proposed a novel integration of Distributed Ledger Technology (DLT) with the TAXII protocol. Leveraging platforms such as Quorum and Hyperledger Fabric, their architecture ensures data immutability. A publish-subscribe middleware (RabbitMQ) facilitates the real-time distribution of cyber threat intelligence, addressing the urgent need for rapid response to advanced cyberattacks on Electrical Power and Energy Systems (EPES). By validating shared data via DLT-stored hashes, the system provides tamper-proof security, non-repudiation, and high reliability, which is critical for safeguarding critical infrastructure.

In a different direction, Preuveneers and Joosen [36] redefined the notion of Indicators of Compromise by introducing a platform that encapsulates machine learning (ML) models as IOCs. This approach overcomes the limitations of short-lived IOCS, such as IP addresses and hash values, by embedding intelligence directly into the detection process. The platform incorporates Ciphertext-Policy Attribute-Based Encryption (CP-ABE) to restrict model access to authorized entities and integrates tools such as MISP, TheHive, and Cortex to facilitate collaborative threat analysis. Furthermore, it classifies potential vulnerabilities, including evasion, poisoning, and model inversion. The study [36] proposes the creation of standardized structures to support the secure sharing of machine learning-based IOCs, positioning this method as a potential standard for dynamic threat detection.

Table 8 summarizes these platforms, highlighting the emerging technologies they incorporate and their reported impact on threat intelligence capabilities.

### 3.4. SOCs and Response Automation

Integrating cyber threat intelligence (CTI) into Security Operations Centers (SOCs) marks a significant evolution in response to the increasing complexity of modern digital threats. While SOCs are crucial in monitoring and incident response, incorporating CTI enables more proactive and informed decision making. Bala Bharathi and Suresh Babu [37] proposed an architecture combining honeypots, Security Information and Event Management (SIEM) systems, and IDS, creating an active defense network. This system not only captures malicious activity but translates it into actionable intelligence. The architecture increases situational awareness and enables real-time responses by luring hackers into honeypot systems. However, the study emphasizes that the effectiveness of automation relies on continuously updating threat models and detection rules.

The emphasis on experimentation in automation highlights the necessity of rigorous and continuous data analysis, particularly given the evolving nature of cyber threats. The comparison between the two studies reveals that the hybrid architecture proposed by [37], combining honeypots, SIEM, and IDS, provides a structured and proactive approach for organizations seeking to enhance their cyber defense capabilities. On the other hand, integrating fuzzy logic offers a practical solution in environments where data uncertainty is constant, enabling adaptive and intelligent threat interpretation. Despite their different focuses, both approaches underline a common requirement: the ongoing refinement and updating of automated tools to keep pace with emerging threats.

The COVID-19 pandemic further amplified the importance of such automation. As Iakovakis et al. [38] observed, the widespread shift to remote work exposed vulnerabilities in existing infrastructures, which cybercriminals exploited through phishing and ransomware. In this context, automation emerged as a critical component of resilient cybersecurity strategies. The study critiqued traditional mitigation tools like vulnerability scanners, which often lack the agility to respond to rapidly changing attack surfaces. A key limitation persists: SOCs must improve their capabilities to filter and prioritize threats as data volumes increase, ensuring that automated outputs are relevant for human analysts.

Ammi [39] emphasized the collaborative dimension of automation by demonstrating the real-time sharing capabilities of the MISP platform. The study argues that the effectiveness of automation extends beyond responsive actions to threats. It depends on how well organizations prepare collectively for them by leveraging shared intelligence. The value of automation is directly related to the quality of the threat intelligence it consumes. Transforming raw data from various sources into timely, actionable insights remains central to this process. However, the complexity of integrating MISP with other tools suggests that automation is not a one-size-fits-all solution but a process that demands coordinated and interoperable systems. As such, large-scale implementations face non-trivial barriers in ensuring seamless integration between different tools and platforms.

In parallel, He et al. [40] explored the role of CTI in response to ransomware attacks within the healthcare sector, drawing lessons from the 2017 WannaCry outbreak, which devastated several hospital systems. Their findings highlighted the limitations of reactive cybersecurity approaches and advocated for proactive, intelligence-driven strategies. This study demonstrated how established methodologies can be tailored to new threat contexts by incorporating the NIST framework. The study also noted a critical limitation: even the most advanced automation systems struggle to predict novel attacks. This underscores the importance of human expertise in interpreting intelligence and guiding strategic decisions.

Khan et al. [41] adopted a proactive approach by emphasizing threat prioritization and behavior analysis through big data analytics. This work illustrates the evolution of SOCs and reflects the need for intelligent filtering mechanisms. Solutions that use honeypots and IDS, combined with tools like IBM QRadar, are designed to detect known threats and effectively distinguish between false threats and real threats. The core challenge addressed is anomaly detection as well as the strategic interpretation and prioritization of threat data. By refining the decision-making process, automation becomes more than a support mechanism, transforming into an active defense partner that enhances the precision of response efforts.

Wang et al. [42] proposed an SOC framework that combines big data and threat intelligence to overcome the constraints of conventional models. Within a unified platform, the architecture collects diverse data streams in real time, including network traffic, security alerts, and system logs. Through multi-perspective analysis, the system identifies specific threats by monitoring services like DNS and SSH and uses natural language processing techniques to extract Indicators of Compromise (IOCs) from unstructured sources, such as blogs and online reports. This enhances situational awareness, ensuring timely identification of internal and external threats. The summary of this section is presented in Table 9 below.

### 3.5. Emerging Threats and Challenges

The cyber threat landscape is evolving rapidly, driven by technological advances such as IoT, blockchain, and artificial intelligence. While these innovations offer significant benefits, they also introduce new vulnerabilities increasingly exploited by sophisticated cybercriminals. The development of cyber threat intelligence is an ongoing battle to keep up with these emerging threats, as attackers continually refine techniques that bypass security systems, exposing gaps in systems previously considered secure. The expansion of Crime-as-a-Service (CaaS) markets on the dark web escalates these risks, requiring a collaborative approach among organizations to comprehend these new tactics’ depth and be prepared to counter them.

The study by Apurv Singh Gautam [43] and his team demonstrates how cyber intelligence can benefit from monitoring dark web forums. These platforms work as a hub for sharing vulnerabilities among hackers, revealing opportunities to understand threat actors’ mindsets and operational patterns. The classification model developed by the authors not only categorizes forum content but also provides insights into the preparation phases of cybercriminals, enabling organizations to anticipate their next steps. This reframes CTI as a strategic asset rather than a purely reactive defense mechanism.

In comparison, Basheer and Alkhatib [46] emphasized the value of dark web monitoring for threat anticipation and acknowledged the associated technical and ethical challenges. These include challenges in maintaining user anonymity and difficulty tracking cryptocurrency transactions. Machine learning and artificial intelligence techniques emerge to combat these threats, enabling more precise predictive analysis, as previously referenced by [43].

Albakri et al. [44] explored the risks associated with CTI sharing, particularly when sensitive information, such as software vulnerabilities or internal business processes, is exposed to adversaries. The study introduced a quantitative risk assessment model that measures both the probability and impact of such exposures, emphasizing the role of trust between sharing entities. Although CTI sharing is critical for strengthening defenses against cyberattacks, the research points out barriers such as a lack of trust, the risk of privacy breaches, and the need for regulatory compliance with the GDPR. To mitigate these risks, anonymization and excluding irrelevant data are recommended, promoting safer collaboration between organizations.

Tatam’s et al. [45] study presented a comprehensive review of cyber threat modeling techniques, focusing on Advanced Persistent Threats (APTs). The study highlighted the role of threat modeling in improving asset visibility and understanding the vulnerabilities commonly exploited by sophisticated attackers. It examined the strengths and limitations of established approaches, including Data Flow Diagrams (DFDs); STRIDE; Attack Trees; and frameworks like MITRE ATT&CK and the Cyber Kill Chain, used to map attackers’ tactics, techniques, and procedures (TTPs). The study concluded that these models often lack automation capabilities, arguing for the integration of machine learning and predictive analysis to improve the detection and mitigation of APTs, increasing the efficiency of threat response.

The study by Javaheri et al. [47] explored cyber threats in the FinTech sector, categorizing them into technological, human, and procedural risks. The authors identified eleven major types of threats, including targeted malware, DDoS attacks, and digital extortion schemes. The study underscored how emerging technologies, such as AI and IoT networks, amplify the threat landscape. Defense mechanisms were explored using machine learning-based detection techniques, highlighting their effectiveness against low-rate and evasive attacks. The study also stressed collaboration between banks, companies, and regulators as essential to strengthening the sector’s resilience, emphasizing the importance of security policies and cybersecurity education to mitigate human failures.

Tounsi and Rais [3] examined the reliance on Indicators of Compromise (IOCs), noting that while they are helpful, their effectiveness is often short-lived due to the constant evolution of threats. Threat intelligence needs to be agile and capable of keeping pace with the rapid evolution of attacks. The authors proposed that automation and machine learning are game changers in how organizations process large volumes of data, allowing more accurate and real-time detection. They suggested standardized threat intelligence formats like STIX and TAXII to enhance interoperability and promote effective intelligence sharing across organizations. Furthermore, the study highlighted emerging threats, including zero-day vulnerabilities and social engineering tactics, which demand more proactive and adaptive detection solutions.

In conclusion, the reviewed literature reinforces the consensus that machine learning plays an essential role in CTI when used to respond to emerging cyber threats. It enhances detection capabilities and enables a shift from reactive to proactive cybersecurity strategies, enhancing resilience in the face of rapidly changing threats. The reviewed studies are presented in Table 10 below, which summarizes these studies by outlining their focus as well as the specific challenges they aim to tackle.

## 4. Discussion

This systematic review demonstrates that the growing sophistication of cyber threats calls for advanced and integrated cyber threat intelligence (CTI) strategies to enhance attack detection and response capabilities. The findings highlight that although advanced technologies are crucial, specific challenges persist.

This discussion explicitly addresses the findings of the 43 reviewed articles to address the research questions highlighted in the introduction. Rather than summarizing individual papers, this section integrates the evidence across the included domains to identify patterns and extract thematic insights.

Effectively integrating CTI into organizational infrastructures is vital to overcoming challenges such as analyzing vast amounts of data and identifying patterns in emerging threats [12,27]. Several sectors prominently benefit from this approach. For instance, in the financial sector, which manages extensive data, machine learning frameworks have proven particularly valuable for uncovering attack patterns, enabling more proactive measures [38]. In healthcare, integrating CTI into incident response, notably after the WannaCry ransomware outbreak, has helped institutions to transition from a reactive stance to a more intelligence-driven, prepared approach. This shift significantly reduced the potential impact of future attacks, a critical factor in a sector where failures can cost lives [40]. Industrial environments and infrastructures have also significantly benefited from CTI implementation. Machine learning techniques combined with balanced datasets have achieved detection accuracy rates of up to 99%. In the energy sector, combining CTI sharing protocols with blockchain technology has strengthened defenses against advanced attacks targeting power systems [35]. However, increased collaboration between organizations is necessary, as smaller companies with limited resources often struggle to fully leverage these strategies due to insufficient high-quality data and a lack of specialists [29,33].

The variety of tools available reflects that no single solution fits all scenarios. Intelligence-sharing platforms, such as the open-source MISP platform, have proven essential, offering significant value by enabling real-time information exchange, especially when integrated with Security Operation Centers (SOCs) [39]. For instance, honeypots effectively enhance security by retaining attackers within a controlled environment—far from critical servers—where attackers believe they are exploiting vulnerabilities yet pose no real threat. This controlled environment enables extensive intelligence gathering, potentially combined with SIEMs and IDS systems, transforming malicious activities into actionable intelligence for analysts [37].

Artificial intelligence-based tools, such as machine learning and deep learning, are widely used for malware and anomaly detection, achieving high success rates. For example, deep learning models applied to IoT environments identified ransomware attacks with over 99% accuracy [44]. Knowledge-based tools, such as cybersecurity knowledge graphs, effectively visualize adversarial tactics and uncover hidden relationships in threat data [27]. The literature thus suggests that combining these tools provides significant advantages in creating deeper, more robust defenses.

The most promising techniques for detecting and preventing cyber threats through CTI are advanced machine learning and behavioral analysis methods. Supervised models, like Random Forest, CNN, and LSTM, have demonstrated high precision in identifying complex threats, particularly when trained on representative datasets. In industrial networks, combining traditional ML methods with data balancing techniques (SMOTE) has achieved nearly 100% accuracy [41]. Deep learning models are particularly effective in detecting novel or disguised attacks. Hybrid architectures can map suspicious behaviors onto knowledge graphs and use neural networks to identify Advanced Persistent Threats (APTs) [41]. Other approaches, such as combining data mining, entity reputation, and time-series forecasting, have successfully predicted recurring attacks and future attack spikes [40]. Reducing reliance is gaining popularity. An example is the use of GANs and BERT for threat detection with minimal labeled data, which significantly reduces the need for extensive datasets [46]. A common observation across these studies is that whereas traditional security systems detect threats based on known attack patterns, current trends increasingly utilize artificial intelligence to proactively predict and identify suspicious behaviors. Nevertheless, these models require constant updates; as cybercriminal techniques continuously evolve [47], failing to update can lead to regression in defense effectiveness.

Despite advancements, several recurring limitations persist in CTI approaches. Foremost among these is the lack of universal standards for threat intelligence data formats. While efforts such as STIX/TAXII standards exist, their limited adoption hinders integration among tools and sharing among organizations [3]. Another critical limitation is the strong dependency on labeled, up-to-date data for training intelligent systems. As threats evolve rapidly, many models quickly lose effectiveness when using outdated or scarce data [42]. Data quality and volume also significantly influence false-positive generation, particularly in scenarios involving large datasets [38]. Furthermore, many organizations remain hesitant to share incidents due to fear of exposure. Wagner [31] highlights the scarcity of trusted platforms that support both manual and automated trust mechanisms. In his study, out of 30 CTI-sharing platforms, only four supported such mechanisms, an insufficient number given the current need.

Studies propose several solutions to address these challenges, such as data standardization through the adoption of frameworks like STIX/TAXII; ensuring privacy and trust via federated learning, which enables collaborative learning without sharing raw data; and employing blockchain and smart contracts to decentralize sharing and ensure data integrity, despite the complexity and associated costs. Additionally, reducing dependency on labeled data through semi-supervised methods like GANs and maintaining continuous updates for both models and teams to keep abreast of emerging threats are crucial measures.

Emerging threats, often driven by technological advances and the exploitation of new vulnerabilities, necessitate transitioning from reactive to proactive defense strategies. The rise of Crime-as-a-Service (CaaS) markets on the dark web has broadened attackers’ access to sophisticated tools and resources, thereby increasing the frequency and complexity of cyber threats [43,46]. Leveraging AI to analyze these underground markets provides valuable insights into adversarial behaviors. Ethical and legal considerations must be carefully managed to prevent privacy violations and regulatory non-compliance. Ethical hacking, therefore, plays a crucial role, allowing organizations to simulate attacks and evaluate their defenses internally.

Another significant concern is the growth of IoT devices combined with the expansion of 5G networks. The extensive number of poorly secured devices and high speeds associated with 5G dramatically expand the attack surface. CTI must adapt to monitor and analyze increasingly distributed data sources [36]. Advanced Persistent Threats (APTs) also continually evolve, employing malware, phishing, and other attack vectors [45].

A common theme among studies is the need to anticipate attacks through continuous CTI updates, which enhance predictive and automated models by integrating real-time monitoring, AI, and cross-sector collaboration.

The findings provide practical relevance through the identification of specific challenges and opportunities across three key areas:

- Technology: CTI systems are typically built on large, labeled datasets and fail to adapt to new attack vectors. The future of CTI technology should focus on data-efficient models such as semi-supervised learning with GANs, integrating AI with knowledge graphs, and ensuring regular model updates to keep pace with the evolving threats.
- Strategies: The traditional use of reactive models is insufficient for today’s dynamic threat landscape. More proactive and intelligence-based strategies are needed that incorporate behavioral analysis, hybrid AI architectures, and continuous monitoring of sources on the “dark-net”.
- Collaborations: CTI’s effectiveness is often limited by a lack of trust and poor standardization. Future directions should support wider adoption of standard frameworks (STIX/TAXII), secure and decentralized platforms (blockchain), and privacy-preserving mechanisms (federated learning) to encourage cross-sector threat information sharing.

### 4.1. Research Challenges

Significant challenges remain despite the considerable progress made in cyber threat intelligence (CTI). The lack of established universal standards is one of the top obstacles to implementing CTI, as these systems must be able to communicate, collaborate, and interact with one another. Without a foundation of common standards, efforts to improve information sharing remain largely unexplored. Additionally, balancing the need for robust security measures with individual privacy protection will continue to be a persistent challenge. Emerging technologies like blockchain can represent promising ways to improve users’ data integrity and privacy.

Another opportunity for development is expanding the design of adaptable machine learning models that do not require the processing of large amounts of labeled data. Cyber threats are evolving faster than ever. Thus, adapting rapidly to limited data will be critical in actively providing a proactive and resilient security posture.

### 4.2. Research Opportunities

Emerging technologies offer potential solutions to current CTI challenges. For example, blockchain has the potential to improve data integrity and privacy by facilitating more secure information sharing with transparency while protecting users’ privacy.

A huge gap in research lies in developing dynamic machine learning models that can be effective, even with limited and unlabeled datasets. This can offer the potential to detect and mitigate threats in real-time, even without comprehensive data being at hand.

Furthermore, the emphasis should be on implementing standards like STIX and TAXII, which should be prioritized to respond to threats faster and implement more effective threat intelligence strategies.

As the number of Internet of Things (IoT) devices increases, and with the imminent expansion of 5G networks, we will have an expanded attack surface that requires more integration of Real-Time Intelligence (RTI) into policies and orchestration platforms that can facilitate engagement and coordination.

To conclude, the future of CTI will depend as much on technological advancements as on the collective commitment of academia, industry, and government. The vision to build a cooperative and resilient digital ecosystem must embrace a greater investment to promote a robust cybersecurity culture and sustained support for research.

## 5. Conclusions

This systematic review highlighted the importance of cyber threat intelligence (CTI) in enhancing cybersecurity measures across various sectors. The integration of CTI into organizational infrastructures enables the anticipation and mitigation of threats, thereby improving the protection of critical assets in an increasingly complex digital landscape. Advanced technologies like machine learning and artificial intelligence have demonstrated efficacy in identifying complex attack patterns and anomalies, thereby promoting a more proactive defense strategy.

However, in order to fully realize the potential of CTI, organizations must consider several key ideas that result from this analysis. Firstly, the adoption of standardized frameworks, such as STIX/TAXII, is essential to improve integration between various CTI tools and facilitate the secure sharing of information between organizations. Subsequently, organizations should invest in semi-supervised learning techniques, such as generative adversarial networks (GANs), to alleviate the prevalent problem of limited labeled threat data, and finally, improved collaboration frameworks, such as federated learning and blockchain technology, should also be considered to improve trust and encourage wider participation in threat intelligence sharing. Future research should prioritize the development of CTI solutions specifically designed for emerging digital environments, including IoT and 5G networks, where the threat landscape is expanding rapidly. Further exploration of automated but adaptable systems that require a minimum of labeled data will also be key to maintaining effectiveness against constantly evolving cyber threats.

## Figures and Tables

**Table 1:** Fundamental technologies and challenges in cyber threat intelligence.

Technology	Main Characteristics	Applications	Challenges
Machine learning	High accuracy in pattern identification	Malware detection, intrusion analysis	Need for large data volumes and constant updates
Blockchain	Immutable records and decentralization	Secure intelligence sharing	Scalability issues and high computational cost
Open-Source Intelligence (OSINT)	Real-time data collection from open sources	Detection of emerging threats, investigation	Variability in data quality and relevance
Metaheuristic optimization	Improved feature selection for analysis	Refinement of detection systems	Configuration complexity and risk of overfitting

**Table 2:** Detailed table of PRISMA 2020 methodology phases.

Phase	Description
Phase 1: Identification	Conduct a systematic search in relevant databases to identify a comprehensive set of potentially relevant studies. The goal is to gather an initial list of research that may be pertinent to the review.
Phase 2: Screening	Evaluate the initial collection of studies by reviewing abstracts and titles to ensure they meet predefined inclusion and exclusion criteria. Irrelevant studies are discarded at this stage.
Phase 3: Eligibility	Perform a detailed assessment of studies that passed the screening to ensure they meet the quality standards required and are directly relevant to the review topic.
Phase 4: Synthesis	Integrate and critically analyze the selected studies to form the foundation of the review’s conclusions. This involves combining data and identifying patterns or gaps in the existing literature.

**Table 3:** Protocol.

Phase	Description
Research Questions	RQ1: How effective are cyber threat intelligence (CTI) strategies in mitigating cyberattacks in different sectors? RQ2: Which tools should be used to combat these attacks efficiently? RQ3: Which analysis methodologies are most effective in detecting and preventing cyber threats through CTI? RQ4: What are the limitations of current CTI approaches, and what strategies can be implemented to overcome them? RQ5: What are the emerging cyber threats that may impact the future development of CTI?
Search Criteria	English language; search keywords
Search Keywords	(“Cyber Threat Intelligence” OR “CTI”) AND (“cybersecurity” OR “information security” OR “cyber attacks” OR “threat mitigation” OR “CTI tools”)
Search Methods	Keyword search, backwards search, forward search
Inclusion Criteria	IC1: Literature with concepts of CTI IC2: Studies that meet the defined keywords and criteria
Exclusion Criteria	EC1: The publication is a duplicate EC2: The paper is not written in English EC3: The study was published before 2018 EC4: The publication record found does not correspond to a full paper EC5: The full version of the article is unavailable EC6: The study does not fit into the scope of the RQs
Quality Criteria	CQ1: Is the article relevant to the field of cyber threat intelligence? CQ2: Does the article evaluate the effectiveness of cyber threat intelligence strategies in mitigating cyberattacks? CQ3: Does the article identify or recommend specific tools to combat cyberattacks using cyber threat intelligence? CQ4: Does the article discuss the most effective analysis methodologies to detect and prevent cyber threats through cyber threat intelligence? CQ5: Does the article highlight the limitations of current cyber threat intelligence approaches and propose solutions to overcome them? CQ6: Does the article explore emerging cyber threats that may impact the development of cyber threat intelligence?
Databases	IEEE Xplore; ScienceDirect; Scopus, Web of Science

**Table 4:** Quality checklist.

QC1	Is the article relevant to the field of cyber threat intelligence?
QC2	Does the article evaluate the effectiveness of cyber threat intelligence strategies in mitigating cyberattacks?
QC3	Does the article identify or recommend specific tools to combat cyberattacks using cyber threat intelligence?
QC4	Does the article discuss the most effective analysis methodologies to detect and prevent cyber threats through cyber threat intelligence?
QC5	Does the article highlight the limitations of current cyber threat intelligence approaches and propose solutions to overcome them?
QC6	Does the article explore emerging cyber threats that may impact the development of cyber threat intelligence?

**Table 5:** Assessment of articles based on the quality criteria (CQ).

Study	QC1	QC2	QC3	QC4	QC5	QC6
Guo et al. [6]	✓	✓	✓	✓	✓	✓
Gao et al. [7]	✓	✓	✓	✓	✓	✓
Saad El Jaouhari et al. [8]	✓	✓	✓	✓	✓	✓
Shin et al. [9]	✓	✓	✓	✓	✓	
Aldhaheri et al. [10]	✓	✓	✓	✓	✓	
Alam et al. [11]	✓	✓	✓	✓	✓	✓
Noor et al. [12]	✓	✓		✓	✓	
Husák et al. [13]	✓	✓		✓	✓	
Tang et al. [14]	✓	✓		✓	✓	
Imran et al. [15]	✓	✓		✓	✓	
Kaur et al. [16]	✓	✓		✓	✓	
Kante et al. [17]	✓	✓		✓	✓	
Homayoun et al. [18]	✓	✓		✓	✓	
Cherqi et al. [19]	✓	✓		✓	✓	
Pour and Bou-Harb [20]	✓	✓		✓	✓	
Xiao [21]	✓	✓		✓	✓	
Huang et al. [22]	✓	✓		✓	✓	
Chang et al. [23]	✓	✓		✓	✓	
K. Zhang et al. [24]	✓	✓		✓	✓	
Park and You [25]	✓	✓		✓	✓	
Trifonov et al. [26]	✓	✓		✓	✓	✓
Gao et al. [27]	✓	✓	✓	✓	✓	✓
Nguyen et al. [28]	✓	✓	✓		✓	
Daou et al. [29]	✓		✓			
X. Zhang et al. [30]	✓	✓	✓		✓	
Wagner et al. [31]	✓				✓	
Bou-Harb et al. [32]	✓	✓			✓	
Van Kranenburg et al. [33]	✓	✓	✓		✓	
Bandara et al. [34]	✓	✓	✓		✓	
Pahlevan et al. [35]	✓		✓		✓	
Preuveneers and Joosen [36]	✓	✓	✓		✓	
Bala Bharathi and Suresh Babu [37]	✓	✓	✓		✓	
Iakovakis et al. [38]	✓	✓			✓	✓
Ammi and Jama [39]	✓	✓	✓		✓	
He et al. [40]	✓	✓			✓	✓
Khan et al. [41]	✓	✓	✓		✓	
Wang et al. [42]	✓	✓	✓		✓	
Gautam et al. [43]	✓			✓	✓	✓
Albakri et al. [44]	✓				✓	✓
Tatam et al. [45]	✓	✓		✓	✓	✓
Basheer and Alkhatib [46]	✓				✓	✓
Javaheri et al. [47]	✓	✓		✓	✓	✓
Tounsi and Rais [3]	✓					

**Table 6:** Summary of CTI integration frameworks.

Study	Framework/Platform	Key Methodologies	Advantages/Limitations
Guo et al. [6]	CTI integration	BERT, deep learning, attention mechanism, Levenshtein variant, CKG	Enhanced entity identification/challenges with continuous data flow
Gao et al. [7]	THREATRAPTOR	NLP, custom query language (TBQL)	Detects subtle patterns/integration with structured data needed
El Jaouhari and Etiabi [8]	FedCTI	Decentralized machine learning in IoT	Protects privacy/consistency across devices
Shin et al. [9]	MITRE ATT&CK Embedding	GloVe embeddings, TMR metric	High accuracy/requires a labeled dataset
Aldhaheri et al. [10]	Deep learning	CNNs, RNNs, LSTMs	Improved detection/requires labeled datasets
Alam et al. [11]	Ladder	BERT, knowledge graphs	Accurate predictions/adapts slowly to new methods

**Table 7:** Summary of behavior analysis and machine learning.

Contribution	Achieved Results	Advantages
Noor et al. [12]	Use of NLP to correlate IOCs	High accuracy in correlating complex events
Husák et al. [13]	Combination of reputation analysis and data mining	Improved prediction of incident peaks
Tang et al. [14]	Application of GNNs for APT analysis	Mapping of suspicious behaviors
Imran et al. [15]	Utilization of SMOTE for data balancing in ICS	Improved detection accuracy in ICS environments
Kaur et al. [16]	Automation of security based on the NIST framework	Automation in incident response
Kante et al. [17]	Ransomware mitigation through honeypot integration	Reduction of false positives in ransomware detection
Homayoun et al. [18]	Combined use of CNN and LSTM for ransomware detection in IoT	99.6% detection rate in IoT devices
Cherqi et al. [19]	GAN-BERT model to reduce reliance on labeled data	Classification with fewer labeled data
Pour & Bou-Harb [20]	Comparison of IDS (Bro vs. Snort) in darknet networks	Better detection of stealthy activities
Xiao [21]	Combination of techniques for malware detection in IoT	High accuracy in IoT malware detection
Huang et al. [22]	Mapping threats using MITRE ATT&CK	Mapping of malicious behaviors
Chang et al. [23]	CySecBERT for CTI information extraction	Improved F1 scores for entity extraction
K. Zhang et al. [24]	BiLSTM-CRF for NER in cybersecurity	Effective capture of entities in CTI texts
Park & You [25]	Development of CTI-BERT specific to cybersecurity	Superior performance in malware detection tasks
Trifonov et al. [26]	Integration of Cyber Kill Chain with threat taxonomy	Structured classification of threats

**Table 8:** Summary of CTI sharing and collection platforms for CTI.

Study	Tools/Platforms	Emerging Technologies and Identified Trends	Impact
Gao et al. [27]	SecurityKG (OSCTI)	Automated intelligence collection using machine learning (ML) and natural language processing (NLP)	Improves data visualization and analysis through a cybersecurity knowledge graph, enhancing threat detection and response efficiency.
Nguyen et al. [28]	Blockchain-based CTI platform	Smart contracts and blockchain for secure intelligence sharing	Ensures privacy and security in CTI exchange, eliminating single points of failure and encouraging high-quality data sharing among stakeholders.
Daou et al. [29]	OSINT on AWS	Collaborative data collection and NoSQL databases	Provides an economical CTI solution for SMEs, enabling malware analysis and threat pattern identification.
X. Zhang et al. [30]	Blockchain with Proof of Reputation (PoR)	Trust-enhancing algorithms for secure CTI sharing	Strengthens inter-organizational trust, preventing the spread of false or malicious data.
Wagner et al. [31]	Comparative analysis of CTI platforms	Trust taxonomy based on sharing activity and stakeholder evaluations	Addresses trust challenges in CTI adoption by proposing a model that ensures data security and safe collaboration across industrial sectors.
Bou-Harb et al. [32]	Data preprocessing models for darknet IPs	Large-scale attack detection using darknet data	Optimizes response to complex cyber threats like DDoS through data processing techniques.
Van Kranenburg et al. [33]	Peer-to-peer networks for SMEs	Graph-based risk information sharing	Promotes cybersecurity resilience among SMEs by securely exchanging threat information.
Bandara et al. [34]	LUUNU platform	Privacy-preserving data sharing and traceability	Enhance collaboration and real-time threat detection, ensuring data integrity and allowing organizations to train models without exposing sensitive information.
Pahlevan et al. [35]	DLT with TAXII	Secure CTI data sharing in the energy sector	Protects critical infrastructures, enabling secure and transparent CTI data exchange to safeguard energy networks against cyber threats.
Preuveneers and Joosen [36]	CP-ABE with MISP and TheHive	Secure machine learning model sharing within security communities	Facilitates collaboration and improves threat detection accuracy by ensuring only authorized access to shared ML models.

**Table 9:** Summary of SOCs and response automation.

Study	Approach/Methodology	Key Findings	Limitations
Bala Bharathi and Suresh Babu [37]	Architecture combining honeypots, SIEM, and IDS.	Created an active defense network where data is transformed into actions. Honeypots attract hackers, increasing surveillance and enabling real-time responses.	Automation relies on frequent updates, indicating that cyber defense strategies must evolve continuously.
Iakovakis et al. [38]	Analysis of remote infrastructure vulnerabilities during COVID-19.	Highlighted deficiencies in traditional mitigation tools, like vulnerability scanners. Emphasized the need for constant reinvention to counter new attack tactics such as phishing and ransomware. Automation must be proactive to remain effective.	Automation can become outdated without proactive updates. Increasing data volumes challenge the effectiveness of automated systems, necessitating better threat filtering and prioritization.
Ammi [39]	Implementation of the MISP platform for real-time threat intelligence sharing.	Effective automation relies on high-quality threat intelligence. Real-time collaboration and data sharing enhance collective preparedness against threats. The integration of MISP with other tools is complex but essential for comprehensive, automated defenses.	Complexity in integrating MISP with other tools indicates that automation requires collaborative processes and cannot rely on a single solution.
He et al. [40]	Applied the NIST model to ransomware response in the healthcare sector.	Proactive use of CTI in shaping prevention strategies can mitigate the impact of attacks and strengthen organizational resilience. Adaptation of established methodologies like NIST to new contexts is effective with high-quality CTI data.	Even with advanced automation, difficulty in accurately predicting the next attack vector maintains the necessity for human oversight and intervention.
Khan et al. [41]	Utilized big data for threat prioritization and behavior analysis, integrating honeypots, IDS, and IBM Q-Radar.	Enhanced ability to distinguish between false positives and real threats. Intelligent prioritization allows security teams to focus on the most urgent threats, making automation a valuable ally in protection rather than just a tool.	Managing and processing large volumes of data effectively to ensure accurate threat prioritization remains challenging.
Wang et al. [42]	Integration of big data with threat intelligence and SOC that collects data and performs multi-perspective real-time analysis. Utilizes natural language processing to extract IOCs from open sources.	The framework identifies specific threats by monitoring DNS, SSH, and network traffic, enhancing the SOC’s real-time response effectiveness.	Dependence on open-source data may limit accuracy in scenarios where information is limited or outdated.

**Table 10:** Summary of emerging threats and challenges.

Study	Main Focus	Methodology/Tools	Challenges Addressed
Gautam et al. [43]	Monitoring dark web forums for threat prediction and neutralization	Machine learning	Difficulty in tracking anonymous transactions and cybercriminal behavior
Basheer and Alkhatib [46]	Analyzing the cybercrime economy on the dark web and hacker network development	Machine learning and data mining	User anonymity and transaction tracking with cryptocurrencies
Albakri et al. [44]	CTI sharing risk assessment, considering the impact of sensitive data disclosure	Quantitative model	Privacy breaches and lack of trust among entities
Tatam et al. [45]	Cyber threat modeling focusing on APTs and TTP mapping frameworks	DFD, STRIDE, Attack Trees, MITRE ATT&CK, Cyber Kill Chain	Lack of automation in threat mapping models
Javaheri et al. [47]	Threat classification in the FinTech sector and defense strategies using machine learning	Machine learning	Increased complexity due to AI and IoT in FinTech
Tounsi and Rais [3]	Dependency on IOCs and automation for emerging threat detection	Automation and ML, STIX, TAXII	Rapid IOC expiration and the emergence of zero-day attacks

## Data Availability

Not applicable.
